# Molecular identification of critically endangered European eels (*Anguilla anguilla*) in US retail outlets

**DOI:** 10.7717/peerj.14531

**Published:** 2023-02-06

**Authors:** Taylor Ely, Nathaniel Patten, Lewis C. Naisbett-Jones, Erin T. Spencer, Demian A. Willette, Peter B. Marko

**Affiliations:** 1 School of Life Sciences, University of Hawai‘i at Mānoa, Honolulu, Hawai‘i, United States of America; 2 Department of Biology, University of North Carolina at Chapel Hill, Chapel Hill, NC, United States of America; 3 Department of Biological Sciences, Florida International University, Miami, FL, United States of America; 4 Biology Department, Loyola Marymount University, Los Angeles, CA, United States of America

**Keywords:** Forensic science, Fish, Food traceability, MtDNA, Cytb, 18S rDNA, Unagi, CITES Appendix-II, Seafood mislabeling

## Abstract

The European eel (*Anguilla anguilla*) has declined by over 90% since the early 1980s and has been listed as critically endangered. Yet, despite strict export bans from the European Union, the European eel is still sold illegally in many countries. Efforts to monitor the trade of European eels have been primarily concentrated in Asian markets where concerningly high rates of European eel have been reported. Comparably fewer studies have assessed the identities of eel samples from the United States (US), despite the obvious implications for eel conservation. To address this knowledge gap, we purchased 137 eel products (134 freshwater eels and three saltwater eels) from grocers, sushi bars, and restaurants in nine states across the US from 2019 to 2021. Seven samples (5.2%) labeled as freshwater eels (or “unagi”) were identified as European eels using a combination of mitochondrial (cytochrome b) and nuclear (18S rRNA) restriction digestion assays, a fast and inexpensive molecular tool for seafood identification that can identify hybrids between European eels (*A. anguilla*) and American eels (*A. rostrata*). No hybrids between European and American eels were found and all seven samples identified with restriction digestion as European eels were confirmed by sequencing of cytochrome b and 18S rRNA. Frequency of European eels in US markets did not significantly correlate with state or retail type. Although illegal eel exports are likely reaching US consumers, the frequency of European eel samples in this study of the US market is much lower than found in other non-European countries.

## Introduction

The European eel (*Anguilla anguilla*) is the most widespread and important single fish stock in Europe (Violi et al., 2015), but is also among the most threatened fisheries species in the world (Dekker, 2008; FAO & ICES, 2006). Several factors, including overfishing, climate change, and habitat destruction have contributed to the decline (Kirk, 2003; Knights, 2003; Brämick, Simon & Fladung, 2006; Winter et al., 2015; Dekker, 2008), with the species currently classified as “critically endangered” on the International Union for Conservation of Nature (IUCN) Red List (Jacoby & Gollock, 2019). To protect the stock, European eels are listed in Appendix-II of the Convention on International Trade in Endangered Species (CITES), requiring permits for all international trade (CITES, 2007). The European Union (EU) also adopted an eel recovery plan (European Union, 2007), banning all import and export of European eels (European Commission, 2010).

Most freshwater eels consumed in the United States (US) are produced by aquaculture facilities in China. Eel aquaculture, however, depends on a supply of wild-caught juvenile eels. High demand and dwindling eel numbers have resulted in an increase in the price of juvenile European eels well in excess of €1,000/kg (Sustainable Eel Group, 2018) and an increase in demand for other anguillids. Consequently, both American (*A. rostrata*) and Japanese (*A. japonica*) eels are considered “endangered” by the IUCN and are also subject to strict national catch quotas and trade restrictions (Nijman, 2015; CITES, 2018; Kaifu et al., 2019). In the United States, states such as New York have implemented policies for the specific conservation of American eel (New York §A10159), whereas new federal legislation has recently passed for broader conservation of species of greatest conservation need (US H.R.2773). Given that the reported production of freshwater eels from farms in China alone exceeds the reported global supply of juvenile eels (CITES, 2017; Kaifu et al., 2019), production is likely fueled by undocumented illegal trade of juvenile eels (Kaifu et al., 2019). Therefore, the retail freshwater eel market likely consists of a mixture of illegally- and legally-traded species, including the critically endangered European eel.

Understanding the scope and scale of trade in illegal seafood products often requires reliable discrimination among closely-related species. Precise seafood identification is also necessary for accurate labeling that allows consumers to avoid seafood from supply chains that contain overfished and threatened stocks (*e.g.*, Jacquet & Pauly, 2007; Logan et al., 2008; Marko et al., 2011). In many instances, however, accurate species-level identification is not possible. In the case of critically endangered European eels, separating specimens based on morphology is difficult for intact specimens and impossible for processed products, which are usually fileted, smoked or roasted, and then either canned or frozen. Retailers and consumers that want to avoid distributing, purchasing, and consuming critically endangered European eels are at a further disadvantage given that the three most economically-important species of freshwater eels sold in the US (*A. anguilla, A. rostrata,* and *A. japonica)* are all traded under the FDA-approved, but uninformative market names of “eel” and “freshwater eel” (US Food and Drug Administration, 2022). The Japanese name “unagi,” considered a vernacular name by the FDA and therefore not a valid market name, is also widely used to describe all species of freshwater eel served in restaurants and sushi bars (Stein et al., 2021).

PCR-based molecular tools can be used to identify and discriminate different seafood species, including freshwater eels (*e.g.*, Richards et al., 2020). These assays rely primarily on amplification and sequencing of mitochondrial DNA (mtDNA), an ideal marker for species discrimination. MtDNA is rapidly evolving, resulting in nucleotide differences that can be used to distinguish closely-related species. Thirteen studies using molecular tools have reported the presence of European eels in restaurants and groceries: across all published studies, European eels are abundant, comprising 59% of retail samples, the vast majority of which were obtained in Hong Kong, Canada, Japan, and South Korea (Nijman & Stein, 2022). Only five studies have included more than 10 freshwater eel samples and only one of those five studies analyzed samples from retail markets outside East Asia (Nijman & Stein, 2022). In the US, the extent of eel mislabelling is based on just four samples of freshwater eel included in peer-reviewed seafood mislabeling studies.

Here, we conducted the first extensive investigation into the species composition of freshwater eels sold in the US. We purchased eel products in nine states and identified them with a DNA-based assay that consisted of a combination of restriction-enzyme digestion and sequencing of PCR products. Screening samples with restriction digestion of PCR products is a rapid and inexpensive way to focus on molecular identification of eels (Stein et al., 2021) while avoiding the more costly and time-consuming strategy of barcoding every sample with DNA sequencing. We screened all samples with restriction-digestion assays for one mtDNA and one nuclear marker to rule out the possibility that eels were misidentified as a consequence of hybridization between co-occurring *A. anguilla* and *A. rostrata* in the North Atlantic (Albert, Jónsson & Bernatchez, 2006; Gagnaire, Normandeau & Bernatchez, 2012; Wielgoss et al., 2014; Nikolic et al., 2020). Our main goal was to determine what fraction of freshwater eels sold in the US are critically endangered European eels, obscured from both law enforcement and consumers. Given that European eels are listed as an Appendix-II species, their export can only be authorized with a CITES export permit or re-export certificate. We sampled freshwater eels sold in the US from 2019 to 2021, a period over which less than 2% of all imported freshwater eel imports were authorized by CITES permits. Therefore, we expected that there should be relatively few European eels detected during the sampling period of our study.

## Methods

### Sample collection

**Figure 1:**
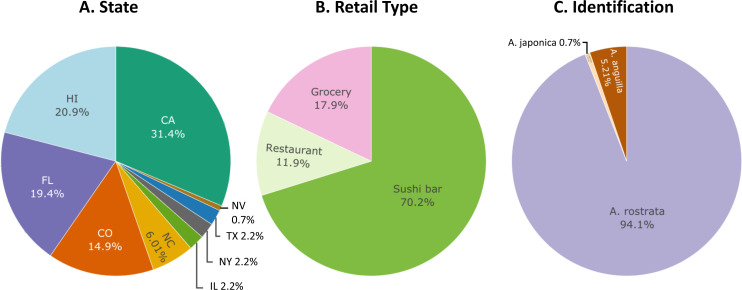
Sampling distribution of 134 freshwater eel samples. Percentages of freshwater eel samples across all (A) states collected in, (B) retail type purchased from, and (C) species identified by this study.

137 eel samples were collected from restaurants, sushi bars, and groceries from November, 2019, to February, 2021 from several major metropolitan areas across the United States, including Hawai‘i, California, Colorado, Florida, Illinois, Texas, Nevada, New York, and North Carolina (Table 1, Fig. 1). In total, 134 of the samples were sold as freshwater eel and labeled as unagi, freshwater eel, and eel, or variations of these labels with additional modifiers describing their preparation (*e.g.*, grilled eel). Three saltwater eel samples, labeled as either anago, yakianago, or saltwater eel were inadvertently purchased but also analyzed. Tissue samples were preserved in 100% ethanol and shipped to the University of Hawai‘i at Mānoa for analysis.

**Table 1:** Origin and identities of samples in this study. The approximate size in base pairs (bp) of visible fragments from the cytochrome b (cytb) and 18S ribosomal RNA (18S) restriction-enzyme assay are provided. Example fragment sizes are shown in Fig. 2. Sizes of bp are approximated from both the DNA ladder and prior knowledge of where the restriction digest proteins cut.

Sample	Date	Retail type	City	State	Labeled as	Cytb assay (bp)	18S rRNA assay (bp)	Identification	Source/Brand	
1	5/18/20	Grocery	Los Angeles	CA	Unagi	280	167, 245	*A. rostrata*	–	
2	5/18/20	Grocery	Los Angeles	CA	Unagi	280	167, 245	*A. rostrata*	–	
3	3/11/20	Grocery	Los Angeles	CA	Unagi Kabayaki	280	167, 245	*A. rostrata*	CFI Premium	
4	6/8/20	Grocery	Los Angeles	CA	Unagi Kabayaki	280	167, 245	*A. rostrata*	CFI Premium	
5	6/8/20	Grocery	Los Angeles	CA	Unagi Kabayaki	280	167, 245	*A. rostrata*	CFI Premium	
6	6/20/20	Grocery	San Gabriel	CA	Unagi	280	167, 245	*A. rostrata*	–	
7	1/2/21	Grocery	Los Angeles	CA	Unagi Kabayaki	280	167, 245	*A. rostrata*	CFI Premium	
8	1/2/21	Grocery	Los Angeles	CA	Unagi Kabayaki	280	167, 245	*A. rostrata*	CFI Premium	
9	1/2/21	Grocery	Los Angeles	CA	Unagi Kabayaki	280	167, 245	*A. rostrata*	CFI Premium	
10	1/2/21	Grocery	Torrance	CA	Unagi Kabayaki	280	167, 245	*A. rostrata*	TTB Seafood and Fish	
11	1/9/21	Grocery	Gardena	CA	Unagi Kabayaki	280	167, 245	*A. rostrata*	Lucky Lakko	
12	1/9/21	Grocery	Gardena	CA	Eel [Unagi]	280	167, 245	*A. rostrata*	JH Seafood Supply Inc.	
13	1/9/21	Grocery	Torrance	CA	Unagi Kabayaki	280	167, 245	*A. rostrata*	Nijiya Market	
14	1/9/21	Grocery	Torrance	CA	OJ Unagi Kabayaki	280	167, 245	*A. rostrata*	OJ	
15	11/16/19	Grocery	Honolulu	HI	Unagi	350	412	***A. anguilla***	CFI	
16	1/29/20	Grocery	Honolulu	HI	Saltwater Eel	280	412	Saltwater eel	Jaeho	
17	11/20/20	Grocery	Honolulu	HI	Unagi Eel	280	167, 245	*A. rostrata*	CFI	
18	11/20/20	Grocery	Honolulu	HI	Unagi Kabayaki	280	167, 245	*A. rostrata*	Uokichi	
19	11/20/20	Grocery	Honolulu	HI	Anago Kabayaki	280	412	Saltwater eel	Kawa Corp	
20	12/8/20	Grocery	Honolulu	HI	Unagi Kabayaki	280	167, 245	*A. rostrata*	Global	
21	12/8/20	Grocery	Honolulu	HI	Chin’s Broiled Eel	280	167, 245	*A. rostrata*	Mister Chin’s	
22	12/8/20	Grocery	Honolulu	HI	Yakianago	280	412	Saltwater eel	Ryujin International	
23	2/15/21	Grocery	Honolulu	HI	Unagi Kabayaki	280	167, 245	*A. rostrata*	Global	
24	7/18/20	Grocery	Durham	NC	Unagi Kabayaki	280	167, 245	*A. rostrata*	Elite	
25	7/18/20	Grocery	Durham	NC	Frozen Broiled Eel	280	167, 245	*A. rostrata*	Rhee Bro’s Inc	
26	7/19/20	Grocery	Cary	NC	Eel	280	167, 245	*A. rostrata*	–	
27	4/15/20	Grocery	Las Vegas	NV		280	167, 245	*A. rostrata*	–	
28	7/5/20	Restaurant	Denver	CO	Unagi	280	167, 245	*A. rostrata*	–	
29	8/4/20	Restaurant	Denver	CO	Unagi	280	167, 245	*A. rostrata*	–	
30	11/18/20	Restaurant	Miami Beach	FL	Unagi Sashimi	280	167, 245	*A. rostrata*	–	
31	11/18/20	Restaurant	Miami Beach	FL	Eel Cucumber Roll	280	167, 245	*A. rostrata*	–	
32	11/18/20	Restaurant	Miami Beach	FL	Unagi (Freshwater Eel)	280	167, 245	*A. rostrata*	–	
33	11/19/20	Restaurant	Ft Lauderdale	FL	Eel Unagi	280	167, 245	*A. rostrata*	–	
34	11/19/20	Restaurant	Ft Lauderdale	FL	Eel	117, 164	412	*A. japonica*	–	
35	11/24/20	Restaurant	Hollywood	FL	Unagi Sushi	280	167, 245	*A. rostrata*	–	
36	11/22/19	Restaurant	Honolulu	HI	Unagi	280	167, 245	*A. rostrata*	–	
37	11/24/19	Restaurant	Honolulu	HI	Eel	280	167, 245	*A. rostrata*	–	
38	11/24/19	Restaurant	Honolulu	HI	Eel	280	167, 245	*A. rostrata*	–	
39	11/24/19	Restaurant	Honolulu	HI	Eel	280	167, 245	*A. rostrata*	–	
40	1/29/20	Restaurant	Honolulu	HI	Unagi	280	167, 245	*A. rostrata*	–	
41	12/7/20	Restaurant	Honolulu	HI	Eel Musubi	280	167, 245	*A. rostrata*	–	
42	8/18/20	Restaurant	Bartlett	IL	Unagi	280	167, 245	*A. rostrata*	–	
43	7/18/20	Restaurant	Durham	NC	Eel Sushi	280	167, 245	*A. rostrata*	–	
44	3/12/20	Sushi bar	Los Angeles	CA	Unagi	280	167, 245	*A. rostrata*	–	
45	3/11/20	Sushi bar	Los Angeles	CA	Freshwater Eel	280	167, 245	*A. rostrata*	–	
46	6/8/20	Sushi bar	Los Angeles	CA	Freshwater Eel	280	167, 245	*A. rostrata*	–	
47	3/18/20	Sushi bar	Los Angeles	CA	Eel Unagi	280	167, 245	*A. rostrata*	–	
48	6/12/20	Sushi bar	Los Angeles	CA	Unagi	280	167, 245	*A. rostrata*	–	
49	6/12/20	Sushi bar	Los Angeles	CA	Freshwater Eel	280	167, 245	*A. rostrata*	–	
50	3/12/20	Sushi bar	Los Angeles	CA	Freshwater Eel	280	167, 245	*A. rostrata*	–	
51	3/11/20	Sushi bar	Los Angeles	CA	Unagi	280	167, 245	*A. rostrata*	–	
52	6/20/20	Sushi bar	Pasadena	CA	Freshwater Eel	280	167, 245	*A. rostrata*	–	
53	3/10/20	Sushi bar	Pasadena	CA	Freshwater Eel	280	167, 245	*A. rostrata*	–	
54	6/12/20	Sushi bar	Los Angeles	CA	Unagi	280	167, 245	*A. rostrata*	–	
55	6/8/20	Sushi bar	Los Angeles	CA	Freshwater Eel	280	167, 245	*A. rostrata*	–	
56	3/11/20	Sushi bar	Los Angeles	CA	Freshwater Eel	280	167, 245	*A. rostrata*	–	
57	9/12/20	Sushi bar	Los Angeles	CA	Freshwater Eel	280	167, 245	*A. rostrata*	–	
58	9/12/20	Sushi bar	Los Angeles	CA	Freshwater Eel	280	167, 245	*A. rostrata*	–	
59	9/13/20	Sushi bar	Los Angeles	CA	Freshwater Eel	280	167, 245	*A. rostrata*	–	
60	9/13/20	Sushi bar	Los Angeles	CA	Freshwater Eel	280	167, 245	*A. rostrata*	–	
61	9/12/20	Sushi bar	Los Angeles	CA	Freshwater Eel	280	167, 245	*A. rostrata*	–	
62	9/13/20	Sushi bar	Los Angeles	CA	Freshwater Eel	280	167, 245	*A. rostrata*	–	
63	9/13/20	Sushi bar	Pasadena	CA	Freshwater Eel	280	167, 245	*A. rostrata*	–	
64	9/13/20	Sushi bar	Los Angeles	CA	Freshwater Eel	280	167, 245	*A. rostrata*	–	
65	1/2/21	Sushi bar	Los Angeles	CA	Unagi	280	167, 245	*A. rostrata*	–	
66	1/2/21	Sushi bar	Los Angeles	CA	Freshwater Eel	280	167, 245	*A. rostrata*	–	
67	1/2/21	Sushi bar	Los Angeles	CA	Freshwater Eel	280	167, 245	*A. rostrata*	–	
68	1/2/21	Sushi bar	Los Angeles	CA	Freshwater Eel	280	167, 245	*A. rostrata*	–	
69	1/2/21	Sushi bar	Los Angeles	CA	Eel	280	167, 245	*A. rostrata*	–	
70	1/3/21	Sushi bar	Glendale	CA	Unagi	350	412	***A. anguilla***	–	
71	1/3/21	Sushi bar	Pasadena	CA	Freshwater Eel	280	167, 245	*A. rostrata*	–	
72	7/7/20	Sushi bar	Denver	CO	Unagi	280	167, 245	*A. rostrata*	–	
73	7/10/20	Sushi bar	Denver	CO	Unagi	280	167, 245	*A. rostrata*	–	
74	7/14/20	Sushi bar	Denver	CO	Unagi	280	167, 245	*A. rostrata*	–	
75	7/14/20	Sushi bar	Denver	CO	Unagi	280	167, 245	*A. rostrata*	–	
76	7/14/20	Sushi bar	Denver	CO	Unagi	280	167, 245	*A. rostrata*	–	
77	7/21/20	Sushi bar	Denver	CO	Unagi	280	167, 245	*A. rostrata*	–	
78	7/21/20	Sushi bar	Denver	CO	Unagi	350	412	***A. anguilla***	–	
79	7/21/20	Sushi bar	Denver	CO	Eel	280	167, 245	*A. rostrata*	–	
80	7/21/20	Sushi bar	Denver	CO	Unagi	280	167, 245	*A. rostrata*	–	
81	7/28/20	Sushi bar	Denver	CO	Unagi	280	167, 245	*A. rostrata*	–	
82	7/28/20	Sushi bar	Denver	CO	Unagi	280	167, 245	*A. rostrata*	–	
83	7/28/20	Sushi bar	Denver	CO	Unagi	280	167, 245	*A. rostrata*	–	
84	7/28/20	Sushi bar	Denver	CO	Unagi	280	167, 245	*A. rostrata*	–	
85	7/28/20	Sushi bar	Denver	CO	Unagi	280	167, 245	*A. rostrata*	–	
86	8/4/20	Sushi bar	Denver	CO	Unagi	280	167, 245	*A. rostrata*	–	
87	8/4/20	Sushi bar	Denver	CO	Unagi	280	167, 245	*A. rostrata*	–	
88	8/4/20	Sushi bar	Denver	CO	Unagi	280	167, 245	*A. rostrata*	–	
89	8/4/20	Sushi bar	Denver	CO	Unagi	280	167, 245	*A. rostrata*	–	
90	5/17/20	Sushi bar	Key Largo	FL	Unagi	280	167, 245	*A. rostrata*	–	
91	5/18/20	Sushi bar	Key Largo	FL	Unagi	280	167, 245	*A. rostrata*	–	
92	5/24/20	Sushi bar	Key Largo	FL	Unagi	280	167, 245	*A. rostrata*	–	
93	5/25/20	Sushi bar	Key Largo	FL	Unagi	280	167, 245	*A. rostrata*	–	
94	5/27/20	Sushi bar	Key Largo	FL	Unagi	280	167, 245	*A. rostrata*	–	
95	6/10/20	Sushi bar	Key Largo	FL	Unagi	280	167, 245	*A. rostrata*	–	
96	11/18/20	Sushi Bar	North Miami	FL	Unagi ”Eel”	280	167, 245	*A. rostrata*	–	
97	11/18/20	Sushi Bar	North Miami	FL	Eel Sashimi	280	167, 245	*A. rostrata*	–	
98	11/18/20	Sushi Bar	North Miami	FL	Freshwater eel	350	412	***A. anguilla***	–	
99	11/19/20	Sushi Bar	Wilton Manors	FL	Unagi	280	167, 245	*A. rostrata*	–	
100	11/19/20	Sushi Bar	Ft Lauderdale	FL	Unagi (Freshwater Eel)	280	167, 245	*A. rostrata*	–	
101	11/19/20	Sushi Bar	Ft Lauderdale	FL	Eel	280	167, 245	*A. rostrata*	–	
102	11/19/20	Sushi Bar	Ft Lauderdale	FL	Eel Nigiri	280	167, 245	*A. rostrata*	–	
103	11/19/20	Sushi Bar	Ft Lauderdale	FL	Eel Unagi	280	167, 245	*A. rostrata*	–	
104	11/19/20	Sushi Bar	Ft Lauderdale	FL	Eel Sashimi	280	167, 245	*A. rostrata*	–	
105	11/19/20	Sushi Bar	Ft Lauderdale	FL	Eel Unagi	280	167, 245	*A. rostrata*	–	
106	11/20/20	Sushi Bar	Hollywood	FL	Eel Unagi	280	167, 245	*A. rostrata*	–	
107	11/20/20	Sushi Bar	Hollywood	FL	Unagi	280	167, 245	*A. rostrata*	–	
108	11/24/20	Sushi Bar	Hollywood	FL	Eel	280	167, 245	*A. rostrata*	–	
109	11/24/20	Sushi Bar	Hollywood	FL	Unagi Eel	280	167, 245	*A. rostrata*	–	
110	11/15/19	Sushi bar	Honolulu	HI	Unagi	280	167, 245	*A. rostrata*	–	
111	11/16/19	Sushi bar	Honolulu	HI	Unagi	280	167, 245	*A. rostrata*	–	
112	11/16/19	Sushi bar	Honolulu	HI	Unagi	280	167, 245	*A. rostrata*	–	
113	11/23/19	Sushi bar	Honolulu	HI	Unagi	350	412	***A. anguilla***	–	
114	11/24/19	Sushi bar	Honolulu	HI	Unagi	280	167, 245	*A. rostrata*	–	
115	11/24/19	Sushi bar	Honolulu	HI	Unagi	280	167, 245	*A. rostrata*	–	
116	1/29/20	Sushi bar	Honolulu	HI	Unagi	280	167, 245	*A. rostrata*	–	
117	1/29/20	Sushi Bar	Honolulu	HI	Unagi	280	167, 245	*A. rostrata*	–	
118	1/29/20	Sushi bar	Honolulu	HI	Unagi	280	167, 245	*A. rostrata*	–	
119	1/29/20	Sushi bar	Honolulu	HI	Unagi	280	167, 245	*A. rostrata*	–	
120	1/29/20	Sushi bar	Honolulu	HI	Unagi	280	167, 245	*A. rostrata*	–	
121	1/29/20	Sushi bar	Honolulu	HI	Eel	350	412	***A. anguilla***	–	
122	1/29/20	Sushi bar	Honolulu	HI	Unagi	280	167, 245	*A. rostrata*	–	
123	1/29/20	Sushi bar	Honolulu	HI	Unagi	280	167, 245	*A. rostrata*	–	
124	1/29/20	Sushi bar	Honolulu	HI	Unagi	280	167, 245	*A. rostrata*	–	
125	12/7/20	Sushi bar	Honolulu	HI	Unagi Roll	280	167, 245	*A. rostrata*	–	
126	8/18/20	Sushi bar	Streamwood	IL	Unagi	350	412	***A. anguilla***	–	
127	8/18/20	Sushi bar	Schaumburg	IL	Unagi	280	167, 245	*A. rostrata*	–	
128	7/18/20	Sushi bar	Durham	NC	Unagi	280	167, 245	*A. rostrata*	–	
129	7/18/20	Sushi bar	Chapel Hill	NC	Eel Nigiri	280	167, 245	*A. rostrata*	–	
130	7/18/20	Sushi bar	Chapel Hill	NC	Eel	280	167, 245	*A. rostrata*	–	
131	7/18/20	Sushi bar	Carrboro	NC	Eel	280	167, 245	*A. rostrata*	–	
132	8/14/20	Sushi bar	Albany	NY	Grilled Eel	280	167, 245	*A. rostrata*	–	
133	8/14/20	Sushi bar	Albany	NY	Unagi	280	167, 245	*A. rostrata*	–	
134	8/5/20	Sushi bar	Albany	NY	Grilled Eel	280	167, 245	*A. rostrata*	–	
135	8/12/20	Sushi bar	New Braunfels	TX	Unagi	280	167, 245	*A. rostrata*	–	
136	8/15/20	Sushi bar	New Braunfels	TX	Unagi	280	167, 245	*A. rostrata*	–	
137	8/9/20	Sushi bar	New Braunfels	TX	Eel Nigiri	280	167, 245	*A. rostrata*	–	

### DNA extraction and polymerase chain reaction

Genomic DNA was extracted from a small piece of cooked skeletal muscle tissue with a 10% Chelex solution (Bio-Rad, Hercules, CA, USA; Walsh, Metzger & Higuchi, 1991). Two gene regions were amplified separately with PCR: 350 bp of mitochondrial cytochrome b (cytb) and approximately 412 bp of 18S ribosomal RNA (18S) using L14841 and H15149 (Kocher et al., 1989) and 18R399 (Struck, Hessling & Purschke, 2002) and Small Subunit F (Englisch & Koenemann, 2001) primers, respectively. These primer pairs are identical to those used in the restriction digestion protocol used here for cytb (Tagliavini, Harrison & Gandolfi, 1995) and 18S (Frankowski & Bastrop, 2010). PCR reaction volumes were 18 µL and included 9 µL of 2x MyTaq Ready Mix (Bioline, Inc.), 6.4 µL of H_2_O, 0.3 µL of each primer (10 mM), and 2 µL DNA (approximately 50 ng/µL). The thermal cycling profile started with an initial denaturation at 94 °C for 3 min followed by 33 cycles of denaturation at 94 °C for 30 s, annealing at 50 °C for cytb and 52 °C for 18S for 30 s, with a slow ramp of 1 °C/s to an extension temperature at 72 °C for 3.5 min. The last cycle was followed by a single extension incubation at 72 °C for 5 min. All samples were successfully amplified. Amplification success was visualized on 1.5% agarose gel.

### Restriction fragment analysis

We identified each sample using a combination of mtDNA and nuclear restriction-enzyme digestion assays. These assays discriminate among *A. anguilla*, *A. rostrata*, and *A. japonica*. Using both mtDNA and nuclear assays allows for detection of potential hybrids and mtDNA introgression between co-distributed *A. anguilla* and *A. rostrata* in the north Atlantic.

**Figure 2:**
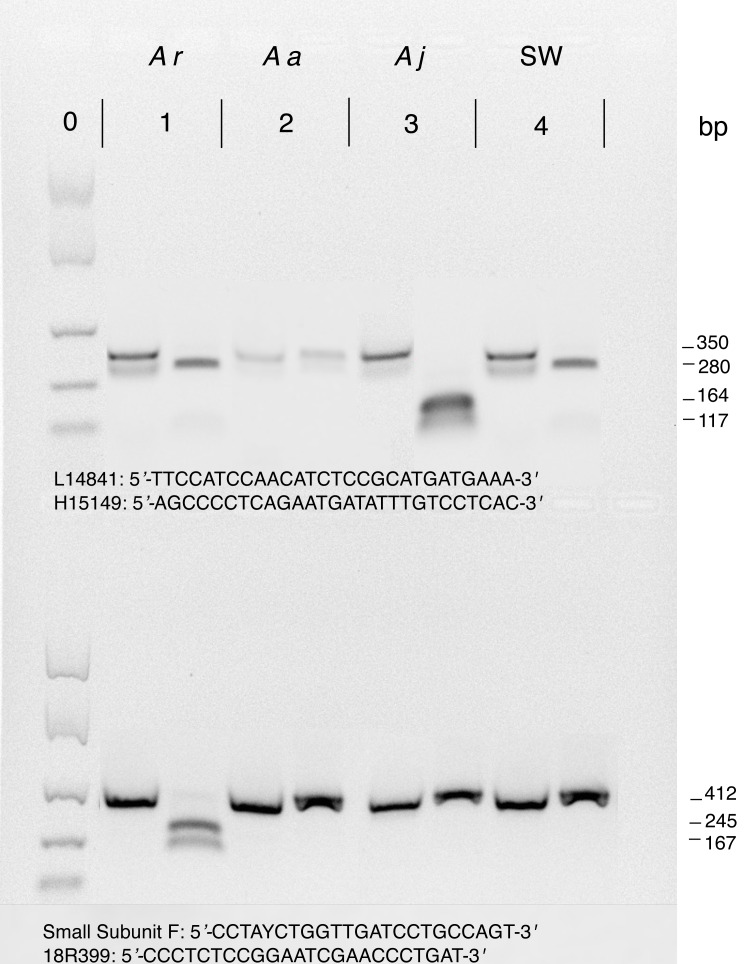
Example restriction digestion of cytb (top) and 18S (bottom) amplicons. For each of four species shown, 5 uL of the uncut PCR product was loaded into the first lane and 5 uL of a digested PCR product was loaded in the second lane. Approximated fragment lengths (bp) are annotated on the right. Lanes and pairs of lanes correspond to (0) EasyLadder (Bioline) size standard (100, 250, 500, 1000, 2000 bp), (1) *A. rostrata,* (2) *A. anguilla*, (3) *A. japonica*, and (4) unknown species of saltwater eel. For cytb, only *A. anguilla* is not cut by HinfII but for 18S only *A. rostrata* is cut by Paul. Primers used for amplification before restriction digestion are below amplicons with L14841 and H15149 for cytb (top) and Small Subunit F and 18R399 for 18S (bottom).

First, 350 bp mitochondrial cytb PCR products were digested with HinfI (Tagliavini, Harrison & Gandolfi, 1995). Cytb amplicons from *A. rostrata* are digested to a single visible 280 bp fragment whereas those from *A. japonica* are digested into two shorter fragments of 117 bp and 164 bp. Cytb amplicons from samples of *A. anguilla* do not contain a HinfI recognition site and therefore remain an uncut fragment of 350 bp after digestion (Fig. 2).

We then digested nuclear 18S amplicons from the same specimens with PauI (Frankowski & Bastrop, 2010). Only *A. rostrata* 18S is cut by PauI, resulting in two visible fragments of 167 bp and 245 bp instead of an undigested 417 bp amplicon (Fig. 2), providing a check of the nuclear background of samples with mtDNA digestion profiles consistent with either *A. rostrata* or *A. anguilla*. To confirm their identity, we then sequenced the cytb and 18S amplicons from every putative sample of *A. anguilla,* samples whose cytb and 18S amplicons were not cut by either enzyme. We also used sequencing to confirm the identities of 15 randomly chosen samples identified by restriction digestions as *A. rostrata* plus all putative samples of *A. japonica*.

18S amplicons from the saltwater eels in our study were not cut by PauI but saltwater eel cytb amplicons were cut by Hinf1 to produce a fragment similar in size to those from *A. rostrata* cytb digestions (Fig. 2), meaning that the restriction-enzyme assay profiles of saltwater eels were consistent with individuals having the nuclear background of either *A. anguilla* or *A. japonica* but possessing the mtDNA of *A. rostrata*. We therefore sequenced cytb from the three saltwater eel samples to verify that they were not freshwater eel hybrids.

Cytb amplicons were digested in 10 uL reactions consisting of 7 uL of the PCR product, 0.2 uL of HinfI, 1 µL CutSmart buffer (New England BioLabs, Ipswich, MA, USA), and 1.8 µL H_2_O. 18S digestions were completed in 10 uL reaction volumes that included 5 µL of the PCR product, 0.2 µL Paul, 1 µL CutSmart buffer, and 3.8 µL H_2_O. For each sample, both the digestion reaction and a negative control (containing only the uncut PCR product) were incubated at 37 °C for one hour followed by enzyme denaturation at 85 °C for 15 min. Digested PCR products and negative controls were assessed side-by-side on 2% agarose gels (Fig. 2).

**Figure 3:**
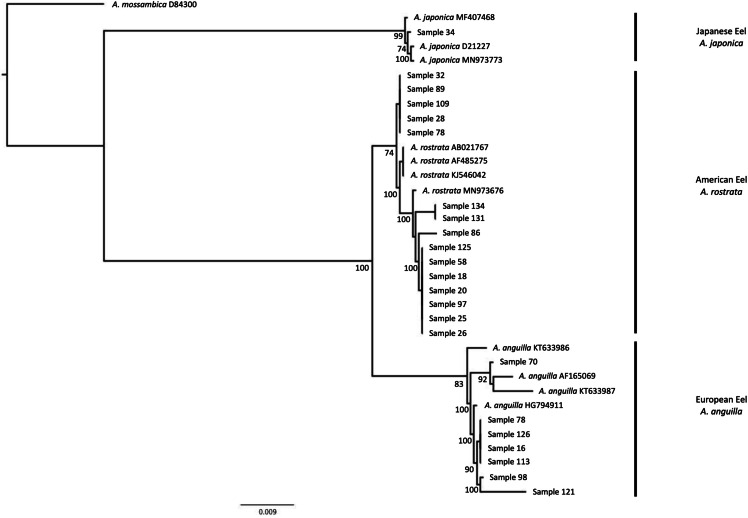
Phylogenetic tree based on cytochrome b region of *Anguilla sp.* samples. Phylogeny created with maximum likelihood methods. Support values are indicated at each node if > 50%. Our samples correspond to sample numbers from Table 1. Multiple randomly selected previously published sequences from GenBank were used as a reference to identify clades to a species-level for *A. japonica*, *A. rostrata*, and *A. anguilla*. *A. mossambica* was used as an outgroup. Samples group correctly to the species that was identified with the restriction digestion assay.

Intact cytb and 18S amplicons were prepared for sequencing by adding 0.1 uL of exonuclease I (New England BioLabs) and 0.9 uL of shrimp alkaline phosphatase (New England Biolabs) and incubated at 37 °C for 30 min followed by enzyme denaturation at 85 °C for 15 min. Amplicons were then sequenced in one direction on an Applied Biosystems 3730XL sequencer at the Advanced Studies in Genomics, Proteomics and Bioinformatics facility at the University of Hawai‘i at Mānoa. Each sequence was compared to published sequences in GenBank with BLAST in Geneious Prime 2020.0.5 (Kearse et al., 2012) with respect to percent pairwise identity, number of top matches, and closest dissimilar hit. A phylogeny of our sequences and reference sequences from GenBank (Accession numbers listed in Fig. 3) was constructed to further validate species identifications. Sequences were aligned with Clustal Omega 1.2.3 in Geneious Prime 2020.0.5 (Kearse et al., 2012). A bootstrapped (*n* = 1000) maximum likelihood (ML) tree was then created from the alignment using the FastTree plugin (Price, Dehal & Arkin, 2009).

### Import and permit records

Import data for freshwater eels (*Anguilla* spp.) was electronically retrieved from the USDA Foreign Agricultural Service’s Global Agricultural Trade System (https://apps.fas.usda.gov/gats/ExpressQuery1.aspx). Records are organized by exporting countries and importing custom districts in the US for years 2018-2021. Records of European eels (*A. anguilla*) imported to the US with CITES permits were electronically-retrieved from the CITES Trade Database (https://trade.cites.org). The records were used to estimate the proportion of freshwater eels that were lawfully-imported European eels.

### Statistical analysis

Percentage of samples sold as freshwater eels but identified as European eels were bootstrapped 1000 times in R v.4.1.0 (Team RC 2021) using the R package *boot* v.1.3.28 (Canty & Ripley, 2021). Generalized linear models (GLM) with a binomial distribution were used to examine possible predictors of freshwater eel sold as *A. anguilla* by state and retail type (grocery, sushi bar, restaurant). Species identification was the response variable. States with low sample sizes, including Illinois, Nevada, New York, and Texas, were removed from the analysis because they did not meet prior assumptions for the GLM. We performed GLMs in R v.4.1.0 using the R package *stats* v.4.1.0 (R Core Team, 2021).

## Results

Among the 137 samples collected, we purchased 27 from grocers (19.7%), 16 from restaurants (11.7%), and 94 from sushi bars (68.6%) (Table 1). Of these, 42 were purchased in California, 20 in Colorado, 26 in Florida, 31 in Hawai‘i, three in Illinois, eight in North Carolina, one in Nevada, three in New York, and three in Texas (Table 1).

Most of our samples were American eels (*A. rostrata*) (Fig. 1). Restriction enzymes cut cytb amplicons from 129 samples to produce a single 280 bp fragment, indicating that they were either American eels or saltwater eels*.* For these 129 samples, 18S amplicons from 126 were cut, identifying them as American eels. We sequenced cytb from the three remaining samples and found that their best matches in GenBank were cytb sequences from saltwater eel genera. None of the saltwater eel samples could be identified to species due to a lack of available reference sequences in GenBank. The three samples that matched saltwater eel sequences were correctly labeled as anago. One additional cytb amplicon was also cut, but to produce two smaller fragments (inferred to be 164 and 117 bp), indicating it was a Japanese eel (*A. japonica*). As expected, 18S from this sample was not cut by PauI, matching the expectation for Japanese eels (Fig. 2).

Cytb amplicons from seven samples were not cut, indicating they were European eels (*A. anguilla*)*.* 18S amplicons from none of these seven were cut by PauI, confirming that they were not American eels with introgressed mtDNA from European eels. The cytb and 18S amplicons from these seven samples were then sequenced; all seven matched GenBank sequences from European eels (Table S1). The 14 sequences were deposited to GenBank (Accession numbers ON815641–ON815654).

The 15 random cytb amplicons identified as American eels (*A. rostrata*) with restriction digest assays all matched GenBank sequences from American eels (Table S1). The cytb amplicon that indicated it was Japanese eel (*A. japonica*) matched to GenBank sequences from Japanese eel (Table S1). The phylogenetic placement of sequences matched results from both restriction digestion assays and searches with BLAST in that all samples clustered to the expected reference sequences for European eel, American eel, and Japanese eel (Fig. 3).

In summary, restriction-enzyme digestion assays and DNA sequencing revealed that seven or 5.2% (*σ* = 2.0%) of our samples labeled as freshwater eels or “unagi” (*n* = 134) were European eels (Fig. 1). The margin of error (95% confidence interval for *n* = 134) for our estimate of 5.2% was ± 3.8%.

Six of the samples sold as freshwater eel were purchased from sushi bars (6.4% of 94 sushi bar samples, *σ* = 2.5%) and one from a grocery (4.2% of 26 grocery samples, *σ* = 4.1%). Three of the European eel samples were purchased in Hawai‘i (10.7% of 28 Hawai‘i samples, *σ* = 5.7%). The other four European eels were each purchased in four different states: California (2.4% of 42 California samples, *σ* = 2.4%), Colorado (5.0% of 20 Colorado samples, *σ* = 4.9%), Florida (3.8% of 26 Florida samples, *σ* = 3.7%), and Illinois (33.3% of three Illinois samples, *σ* = 27.3%). Neither state (X^2^ = 3.70, *N* = 123, *df* = 4, *p* = 0.22) nor retail type (X^2^ = 1.69, *N* = 123, *df* = 2, *p* = 0.38) was a significant predictor of European eels in the GLM.

Over the course of our study (2019–2021) 16,795,248 kg of freshwater eel imports to the U.S were reported to the USDA (Table S2), which included 265,500 kg of legally-imported European eels with CITES permits (Table S3). Therefore, 1.6% of the freshwater eels imported into the US were lawfully-exported European eels.

## Discussion

High frequencies of critically endangered European eel have been reported in many global markets, yet little information exists about the frequency of European eels sold in the US. In our study, we completed the most comprehensive DNA analysis of freshwater eel samples in the US market to date, comprising 134 samples across nine states. We found only seven European eels among the 134 samples (5.2%) sold as freshwater eel, most commonly labeled as “unagi.” These results demonstrate that although making up only ∼5% of the freshwater eel sold in the US, critically endangered European eels are sold under uninformative names, such as freshwater eel and unagi. We also found that neither retail type nor state were related to the frequency of European eel, thus we cannot infer any particular pattern in finding critically endangered European eels across the US and among retailers.

Unlike previous molecular identification studies of freshwater eels, our use of both nuclear and mtDNA markers allowed us to rule out the possibility that some samples with mtDNA matching *A. anguilla* were actually *A. rostrata* individuals with introgressed *A. anguilla* mtDNA. Although hybrids between *A. anguilla* and *A. rostrata* have been reported almost exclusively from Iceland (Avise et al. 1990; Albert, Jónsson & Bernatchez, 2006), the frequency of first and subsequent generation hybrids in Iceland populations (15.5%, Albert, Jónsson & Bernatchez, 2006) greatly exceeds the frequency of *A. anguilla* in our study. However, because each individual with a mtDNA genome matching *A. anguilla* had a matching nuclear background from *A. anguilla*, we conclude that our results were not biased by hybridization and mtDNA introgression and that previous molecular identification studies of freshwater eels are likely similarly unbiased.

Given the low rate of mislabeling, it is unclear if any of the eels we purchased were illegally exported to the US. Over the course of our study, approximately 1.6% of all freshwater eels (by weight) imported into the US were legally imported with CITES documentation. The freshwater eel products we purchased (starting in late 2019) could have been imported to the US earlier and stored frozen, but inclusion of data from 2018 results in a similar, but slightly lower expected frequency of European eels (1.3%). That said, over the course of our study from 2019 to 2021, the expected frequency of legally-exported European eels (1.6%) falls slightly within the 95% confidence interval (1.4 - 9.0%) for the frequency of European eels detected in our study. Because we found such a low rate of European eels, we elected to sequence cytb from 15 randomly selected samples that were identified as *A. rostrata* by restriction digestion, confirming that none were European eels.

The frequency of European eels in our study was much lower than in several other recent molecular identification studies of freshwater eels. For example, the frequency of European eels in our study was radically lower than in a study of Hong Kong groceries and wet markets that found that 45% of eels were European eels (Richards et al., 2020). Similarly, a review of 13 studies from nine countries outside of Europe found that, on average, 59% of freshwater eels were European eels (Nijman & Stein, 2022); the meta-analysis included unpublished data from Canada, where 36% of freshwater eels were European eels. In contrast, European eels were relatively scarce in a recent survey of 108 eel samples in Europe which only found one (<1%) European eel (Stein et al., 2021).

One explanation for the high variation in the frequency of European eels at the global scale is that their abundance reflects the relative strength and enforcement of national and international trade regulations (Stein et al., 2021), suggesting that enforcement of trade restrictions on European eel is substantially more effective in the US and Europe. The low frequency of European eels in our more recent study might also reflect temporal variation in natural juvenile eel supply and subsequent shifts in the supply of *A. anguilla* and *A. rostrata* juveniles (Shiraishi & Crook, 2015; ICES, 2018; Richards et al., 2020). Before our study, only four samples of freshwater eel in the US market (sampled in 2014 and 2016) have been included in molecular identification studies (Khaksar et al., 2015; Wallstrom et al., 2020). Although three of those four samples were identified as *A. anguilla*, the low sample size prevented assessment of the frequency of European eel in the US market prior to our study. Our data are likely insufficient to detect any variance in the frequency of European eels among states within the US, which also makes national averages challenging to interpret (Wallstrom et al., 2020). Given that the frequency of European eels among states in the US was greatest in Hawai‘i (10.7%), where per capita seafood consumption is twice that on the mainland US (Geslani et al., 2012), the demand for seafood may cause retailers to purchase seafood from high volume suppliers that may obtain a larger proportion of illegally-sourced juvenile European eels.

We were unable to assess any associations between the suppliers of mislabeled European eels and the frequency of European eels. Most freshwater eels consumed in the US are at sushi bars and restaurants, which was reflected in our sampling in which we obtained one sample from as many different vendors as possible. None of the samples purchased in sushi bars and restaurants included information about the origin of eels, so no sourcing information is provided for those vendors (Table 1). We did return to those Honolulu vendors that sold samples identified as European eel and purchased additional samples (*n* = 4, Table 1, sample numbers 17,18, 41, 125). However, resampling detected no additional samples that were European eels, indicating that retail vendors are not consistently buying and receiving European eels. Resampling grocery samples with the same branding produced the same outcome, indicating that distributors are likely obtaining eels from multiple sources or that European and non-European eels are mixed at the farming stage of the supply chain.

Even if legally-exported to the US, several factors cause retailers and consumers to unknowingly sell and consume critically endangered European eels in the US First, Country of Origin Labeling (COOL) in the US only requires that the last country in the supply chain be listed as the country of origin for any seafood product (7 USC §1638a). Even though the vast majority of freshwater eels sold in the US originate from the Atlantic Ocean, their country of “origin” is typically China, where juvenile eels are imported, raised, processed, and exported. Second, even if the true country of origin was required by COOL, restaurants and sushi bars, where most freshwater eels are sold in the US (as well as processed eel products sold in groceries) are all exempt from COOL (7 CFR §60.119). Lastly, “eel” is the US FDA Acceptable Market Name for each of *A. anguilla*, *A. australis*, *A. japonica*, and *A. rostrata* (US FDA CFR 101.18c); *A. rostrata* may also be sold as “freshwater eel.” Although the majority of freshwater eels in our study (82 of 134) were sold as “unagi,” existing labeling conventions provide no specificity for retailers and consumers that want to avoid purchasing and consuming critically endangered European eels. Given that all of the economically important species of *Anguilla* are in decline and in the absence of greater traceability measures, the only recourse for consumers that want to avoid endangered species is to not consume any freshwater eels.

Lastly, our analytical approach was relatively inexpensive and can be completed by personnel with knowledge of only basic molecular methods. The cost of standard molecular barcoding with Sanger sequencing of PCR products, the leading method for seafood identification (Fernandes, Amaral & Mafra, 2021), was US$7.97 per sample (including sequencing costs of US$3.50 per reaction for each of cytb and 18S; US$4.47 if only cytb was sequenced). Our use of restriction-enzyme assays greatly reduced the amount of DNA sequencing, the most expensive part of the analysis, to only those samples that could not be positively identified (by cutting a PCR product) with the restriction-enzyme assays as either *A. rostrata* or *A. japonica*. This approach reduced the analytical (PCR and gel-electrophoresis) costs of our study to US$1.15 per sample. Many researchers may still opt for DNA sequencing of some samples so that sequence records may be submitted to GenBank: in our study, the additional sequencing (cytb and 18S) costs for those samples identified as European eels by the restriction-enzyme assays resulted in a cost of US$1.56 per sample (averaged across all 134 freshwater eel samples). Quantitative PCR is more rapid than either restriction-enzyme assays (given the elimination of gel-visualization of PCR products), but has a higher cost ($2.44 per sample) and requires a larger initial investment in a specialized thermal cycler plus greater expertise to design experiments and interpret results. Our combination of a standard Chelex extraction with PCR and restriction digestion is also relatively quick such that our entire protocol can rule out the presence of European eels in seafood samples in less than a day without any DNA sequencing. Bulk extraction and restriction digestion of multiple samples could also be used to increase sample throughput, making this an effective method for identification of potentially illegally-traded species that cannot be visually identified, such as European eel products.

## Conclusions

Using a relatively inexpensive and rapid method of analysis, we found that freshwater eel products purchased from retailers in the US included a low frequency (5.2%) of critically endangered European eels. The frequency of European eels in our study did not fall outside the expected frequency of European eels based on USDA import data and CITES export permit data, suggesting that relatively few illegally exported eels are sold in the US. The low frequency of European eels in our study could reflect temporal variation in juvenile eel supply but might also reflect the relative strength and enforcement of national and international trade regulations. Although at a relatively low frequency in the US, critically endangered European eels are nevertheless sold under uninformative names along with other species of anguilids, preventing US consumers from avoiding one of the most endangered commercial seafood products in the world.

##  Supplemental Information

10.7717/peerj.14531/supp-1 Supplemental Information 1 GenBank matches from BLAST Sample number corresponds to those in Table 1. The top result from BLAST using GenBank is listed along with its accession number, the corresponding percent identity, and how many of the top matches were the same species. The next closest matching sequence is listed as top GenBank dissimilar hit along with its accession number and percent identity. Cytb amplicons (boldface lines) were very useful for species identification with a large number of top matches often at 100% identity and the next closest species much lower in percent identity with no subsequent matches to the correct species after that. However, 18S could not always differentiate between *Anguilla anguilla* and *Anguilla japonica* (sample 70 and 121), similar to the restriction digest assays. All top GenBank hits match results found by restriction digestion assays and phylogeny topology.

10.7717/peerj.14531/supp-2 Supplemental Information 2 US imports of freshwater eels Data from USDA Foreign Agricultural Service Global Agricultural Trade System for 2018–2021 documenting imported eel (*Anguilla spp.*) in kilograms from each country into each US customs district that received eel during this period. Data accessed February 22, 2022 (https://apps.fas.usda.gov/gats/ExpressQuery1.aspx).

10.7717/peerj.14531/supp-3 Supplemental Information 3 A. rostrata and A. japonica DNA sequences

10.7717/peerj.14531/supp-4 Supplemental Information 4 CITES data for European eels (*Anguilla anguilla*) exported to the U.S Appendix-II export data from 2018-2021. Data accessed 01 June 2022 (https://trade.cites.org).

10.7717/peerj.14531/supp-5 Supplemental Information 5 Anguilla anguilla DNA sequences

## Additional Information and Declarations

### Field Study Permissions

The following information was supplied relating to field study approvals (i.e., approving body and any reference numbers): No permit was required for samples obtained from retail outlets.

### Data Availability

The following information was supplied regarding data availability: The sequences are available at GenBank: ON815641–ON815654.
