# Supplementary material for: Molecular identification of critically endangered European eels (Anguilla anguilla) in US retail outlets
Source: PeerJ. 2023 Feb 6;11:e14531. doi: 10.7717/peerj.14531 (PMC9910185; doi:10.7717/peerj.14531)
Supplement: Supplemental Information 1 — Sample number corresponds to those in Table 1. The top result from BLAST using GenBank is listed along with its accession number, the corresponding percent identity, and how many of the top matches were the same species. The next closest matching sequence is listed as top GenBank dissimilar hit along with its accession number and percent identity. Cytb amplicons (boldface lines) were very useful for species identification with a large number of top matches often at 100% identity and the next closest species much lower in percent identity with no subsequent matches to the correct species after that. However, 18S could not always differentiate between Anguilla anguilla and Anguilla japonica (sample 70 and 121), similar to the restriction digest assays. All top GenBank hits match results found by restriction digestion assays and phylogeny topology. [file peerj-11-14531-s001.docx]

**Table S1 GenBank matches from BLAST.** Sample number corresponds to those in Table 1. The top result from BLAST using GenBank is listed along with its accession number, the corresponding percent identity, and how many of the top matches were the same species. The next closest matching sequence is listed as top GenBank dissimilar hit along with its accession number and percent identity. Cytb amplicons (boldface lines) were very useful for species identification with a large number of top matches often at 100% identity and the next closest species much lower in percent identity with no subsequent matches to the correct species after that. However, 18S could not always differentiate between *Anguilla anguilla* and *Anguilla japonica* (sample 70 and 121), similar to the restriction digest assays. All top GenBank hits match results found by restriction digestion assays and phylogeny topology.

| Sample | Marker | Top GenBank hit | Accession | % identity | Number top matches of same species | Top GenBank  dissimilar hit | Accession | % identity |
| --- | --- | --- | --- | --- | --- | --- | --- | --- |
| **16** | **cytb** | ***Anguilla anguilla*** | **NC_006531** | **100** | **120** | ***Anguilla rostrata*** | **MT667251** | **98.9** |
| 16 | 18S | *Anguilla anguilla* | FM946070 | 100 | 2 | *Anguilla luzonensis* | HQ197929 | 99.9 |
| **70** | **cytb** | ***Anguilla anguilla*** | **KJ564267** | **100** | **120** | ***Anguilla rostrata*** | **MT667251** | **98.9** |
| 70 | 18S | *Anguilla anguilla* | FM946070 | 100 | 2 | *Anguilla japonica* | LC464086 | 100 |
| **78** | **cytb** | ***Anguilla anguilla*** | **NC_006531** | **100** | **120** | ***Anguilla rostrata*** | **MT667251** | **98.9** |
| 78 | 18S | *Anguilla anguilla* | FM946070 | 99.9 | 2 | *Anguilla luzonensis* | HQ197929 | 99.8 |
| **98** | **cytb** | ***Anguilla anguilla*** | **NC_006531** | **96.3** | **120** | ***Anguilla rostrata*** | **MT667251** | **95.2** |
| 98 | 18S | *Anguilla anguilla* | FM946070 | 99.9 | 2 | *Anguilla luzonensis* | HQ197929 | 99.7 |
| **113** | **cytb** | ***Anguilla anguilla*** | **NC_006531** | **100** | **120** | ***Anguilla rostrata*** | **MT667251** | **98.9** |
| 113 | 18S | *Anguilla anguilla* | FM946070 | 100 | 2 | *Anguilla luzonensis* | HQ197929 | 99.9 |
| **121** | **cytb** | ***Anguilla anguilla*** | **NC_006531** | **96** | **120** | ***Anguilla rostrata*** | **MT667251** | **93.7** |
| 121 | 18S | *Anguilla anguilla* | FM946070 | 99.9 | 2 | *Anguilla japonica* | FJ231895 | 99.9 |
| **126** | **cytb** | ***Anguilla anguilla*** | **NC_006531** | **100** | **120** | ***Anguilla rostrata*** | **MT667251** | **98.9** |
| 136 | 18S | *Anguilla anguilla* | FM946070 | 99.8 | 2 | *Anguilla luzonensis* | HQ197929 | 99.7 |
| **18** | **cytb** | ***Anguilla rostrata*** | **KJ564217** | **100** | **136** | ***Anguilla japonica*** | **KJ948424** | **99.3** |
| **20** | **cytb** | ***Anguilla rostrata*** | **KJ564217** | **100** | **136** | ***Anguilla japonica*** | **KJ948424** | **99.3** |
| **25** | **cytb** | ***Anguilla rostrata*** | **KJ564217** | **100** | **136** | ***Anguilla japonica*** | **KJ948424** | **99.3** |
| **26** | **cytb** | ***Anguilla rostrata*** | **KJ564217** | **100** | **136** | ***Anguilla japonica*** | **KJ948424** | **99.3** |
| **28** | **cytb** | ***Anguilla rostrata*** | **MT667251** | **99.4** | **63** | ***Anguilla japonica*** | **KJ948424** | **98.7** |
| **32** | **cytb** | ***Anguilla rostrata*** | **MT667251** | **100** | **136** | ***Anguilla japonica*** | **KJ948424** | **99.2** |
| **58** | **cytb** | ***Anguilla rostrata*** | **KJ564217** | **100** | **136** | ***Anguilla japonica*** | **KJ948424** | **99.3** |
| **78** | **cytb** | ***Anguilla rostrata*** | **MT667251** | **98.8** | **63** | ***Anguilla japonica*** | **KJ948424** | **98.1** |
| **86** | **cytb** | ***Anguilla rostrata*** | **KJ564213** | **99.4** | **63** | ***Anguilla japonica*** | **KJ948424** | **98.7** |
| **89** | **cytb** | ***Anguilla rostrata*** | **MT667251** | **100** | **134** | ***Anguilla japonica*** | **KJ948424** | **99.3** |
| **97** | **cytb** | ***Anguilla rostrata*** | **KJ564217** | **100** | **136** | ***Anguilla japonica*** | **KJ948424** | **99.3** |
| **109** | **cytb** | ***Anguilla rostrata*** | **MT667251** | **100** | **136** | ***Anguilla japonica*** | **KJ948424** | **99.2** |
| **125** | **cytb** | ***Anguilla rostrata*** | **KJ564217** | **100** | **136** | ***Anguilla japonica*** | **KJ948424** | **99.3** |
| **131** | **cytb** | ***Anguilla rostrata*** | **KJ564195** | **100** | **136** | ***Anguilla japonica*** | **KJ948424** | **99.2** |
| **134** | **cytb** | ***Anguilla rostrata*** | **KJ564195** | **100** | **136** | ***Anguilla japonica*** | **KJ948424** | **99.2** |
| **34** | **cytb** | ***Anguilla japonica*** | **MH050933** | **100** | **70** | ***Anguilla reinhardti*** | **D84301** | **96.9** |
