# Supplementary material for: Molecular identification of critically endangered European eels (Anguilla anguilla) in US retail outlets
Source: PeerJ. 2023 Feb 6;11:e14531. doi: 10.7717/peerj.14531 (PMC9910185; doi:10.7717/peerj.14531)
Supplement: Supplemental Information 2 — Data from USDA Foreign Agricultural Service Global Agricultural Trade System for 2018–2021 documenting imported eel (Anguilla spp.) in kilograms from each country into each US customs district that received eel during this period. Data accessed February 22, 2022 (https://apps.fas.usda.gov/gats/ExpressQuery1.aspx). [file peerj-11-14531-s002.docx]

**Table S2** **U.S. imports of freshwater eels**. Data from USDA Foreign Agricultural Service Global Agricultural Trade System for 2018-2021 documenting imported eel (*Anguilla spp.*) in kilograms from each country into each US customs district that received eel during this period. Data accessed February 22, 2022 (https://apps.fas.usda.gov/gats/ExpressQuery1.aspx).

| U.S. Custom District | Exporter | 2018 | 2019 | 2020 | 2021 |
| --- | --- | --- | --- | --- | --- |
| New York, NY | China | 2,162,543 | 1,894,715 | 1,187,991 | 3,585,462 |
| New York, NY | Taiwan | 36,986 | 74,707 | 42,762 | 88,114 |
| New York, NY | Indonesia | 0 | 0 | 0 | 54,684 |
| New York, NY | Bangladesh | 38,365 | 48,837 | 35,675 | 93,233 |
| New York, NY | Morocco | 0 | 0 | 0 | 49,854 |
| New York, NY | Dominican Republic | 0 | 0 | 11,407 | 42,565 |
| New York, NY | Thailand | 55,009 | 78,404 | 42,176 | 27,481 |
| New York, NY | Korea, South | 1,877 | 400 | 11,760 | 19,663 |
| New York, NY | Vietnam | 30,154 | 3,314 | 20,111 | 31,163 |
| New York, NY | India | 0 | 0 | 0 | 29,000 |
| New York, NY | Japan | 9,849 | 10,496 | 10,585 | 2,923 |
| New York, NY | Portugal | 2,982 | 6,164 | 3,838 | 2,300 |
| New York, NY | Burma | 0 | 0 | 0 | 2,200 |
| New York, NY | Spain | 0 | 0 | 572 | 157 |
| New York, NY | Pakistan | 0 | 0 | 0 | 1,000 |
| New York, NY | Ukraine | 0 | 0 | 30,289 | 760 |
| New York, NY | New Zealand | 41,707 | 36,123 | 21,068 | 0 |
| New York, NY | Poland | 0 | 0 | 1,284 | 0 |
| New York, NY | Russia | 0 | 0 | 4,920 | 0 |
| Los Angeles, CA | China | 3,455,508 | 2,702,891 | 1,058,383 | 2,751,591 |
| Los Angeles, CA | Thailand | 0 | 27,216 | 109,064 | 136,080 |
| Los Angeles, CA | Japan | 28,405 | 71,180 | 58,587 | 75,798 |
| Los Angeles, CA | Vietnam | 32,126 | 25,640 | 47,461 | 82,689 |
| Los Angeles, CA | Korea, South | 20,544 | 53,499 | 33,042 | 53,327 |
| Los Angeles, CA | Taiwan | 47,984 | 47,064 | 51,692 | 33,812 |
| Los Angeles, CA | Hong Kong | 0 | 20,800 | 90,340 | 10,000 |
| Los Angeles, CA | Ecuador | 0 | 0 | 0 | 10,000 |
| Los Angeles, CA | Burma | 0 | 0 | 0 | 3,780 |
| Los Angeles, CA | Peru | 0 | 0 | 0 | 1,232 |
| Los Angeles, CA | Spain | 58 | 0 | 0 | 820 |
| Los Angeles, CA | New Zealand | 0 | 0 | 0 | 990 |
| Los Angeles, CA | Indonesia | 0 | 0 | 785 | 252 |
| Los Angeles, CA | Portugal | 1,951 | 865 | 0 | 0 |
| Chicago, IL | China | 82,730 | 64,815 | 73,870 | 131,300 |
| Chicago, IL | Spain | 0 | 0 | 0 | 61 |
| Chicago, IL | Japan | 0 | 2,227 | 1,722 | 538 |
| Chicago, IL | Peru | 0 | 12,169 | 0 | 0 |
| Boston, MA | Thailand | 0 | 0 | 68,040 | 163,296 |
| Boston, MA | Portugal | 2,207 | 3,824 | 8,867 | 8,648 |
| Boston, MA | Bangladesh | 9,534 | 14,492 | 1,800 | 0 |
| Portland, ME | Canada | 29,199 | 55,237 | 73,969 | 79,503 |
| San Francisco, CA | Thailand | 0 | 13,608 | 27,216 | 27,216 |
| San Francisco, CA | China | 29,018 | 0 | 6,033 | 22,000 |
| San Francisco, CA | Taiwan | 18,177 | 11,478 | 20,836 | 17,012 |
| San Francisco, CA | Vietnam | 0 | 2,302 | 3,659 | 8,364 |
| San Francisco, CA | Japan | 2,447 | 8,846 | 684 | 0 |
| San Francisco, CA | Korea, South | 455 | 0 | 0 | 0 |
| San Francisco, CA | Portugal | 0 | 0 | 187 | 0 |
| San Francisco, CA | Ukraine | 0 | 0 | 8,724 | 0 |
| Charlotte, NC | China | 0 | 62,600 | 60,000 | 60,000 |
| Honolulu, HI | China | 8,500 | 0 | 4,600 | 11,041 |
| Honolulu, HI | Japan | 8,363 | 9,095 | 6,730 | 6,321 |
| Honolulu, HI | Korea, South | 798 | 50 | 0 | 120 |
| Baltimore, MD | China | 8,880 | 22,780 | 2,560 | 15,632 |
| Baltimore, MD | Taiwan | 1,209 | 4,293 | 6,001 | 528 |
| Baltimore, MD | Ecuador | 8,784 | 0 | 0 | 0 |
| Baltimore, MD | Korea, South | 0 | 969 | 0 | 0 |
| Baltimore, MD | Vietnam | 0 | 4,096 | 0 | 0 |
| Buffalo, NY | Canada | 34,361 | 4,139 | 1,043 | 15,782 |
| Buffalo, NY | Malaysia | 0 | 0 | 0 | 10,517 |
| Miami, FL | China | 28,600 | 36,000 | 18,900 | 88,000 |
| Miami, FL | Japan | 5 | 0 | 0 | 10 |
| Miami, FL | Madagascar | 0 | 0 | 19,512 | 0 |
| Miami, FL | Spain | 1,112 | 1,273 | 1,500 | 0 |
| Charleston, SC | China | 0 | 0 | 0 | 20,000 |
| Norfolk, VA | Thailand | 0 | 0 | 0 | 13,608 |
| Norfolk, VA | China | 7,087 | 30,996 | 0 | 0 |
| Norfolk, VA | Spain | 0 | 61 | 21 | 0 |
| Tampa, FL | China | 9,000 | 0 | 0 | 44,000 |
| Savannah, GA | Korea, South | 0 | 0 | 2,162 | 915 |
| Savannah, GA | China | 8,437 | 2,267 | 0 | 0 |
| Detroit, MI | Canada | 7,883 | 22,088 | 3,698 | 1,660 |
| Seattle, WA | Taiwan | 1,800 | 2,160 | 3,240 | 1,800 |
| Seattle, WA | China | 0 | 28,000 | 9,500 | 0 |
| Houston-Galveston, TX | Vietnam | 1,225 | 0 | 2,458 | 1,961 |
| Houston-Galveston, TX | Netherlands | 0 | 11,000 | 0 | 0 |
| Houston-Galveston, TX | Spain | 0 | 111 | 0 | 0 |
| Great Falls, MT | Spain | 0 | 51 | 0 | 0 |
| St. Louis, MO | Vietnam | 0 | 8,788 | 0 | 0 |
| San Juan, Puerto Rico | Spain | 0 | 481 | 550 | 0 |
| **Total** |  | 6,275,859 | 5,542,611 | 3,311,874 | 7,940,763 |
