# Supplementary material for: Molecular identification of critically endangered European eels (Anguilla anguilla) in US retail outlets
Source: PeerJ. 2023 Feb 6;11:e14531. doi: 10.7717/peerj.14531 (PMC9910185; doi:10.7717/peerj.14531)
Supplement: Supplemental Information 4 — Appendix-II export data from 2018-2021. Data accessed 01 June 2022 (https://trade.cites.org). [file peerj-11-14531-s004.docx]

**Table S3** **CITES data for European eels (*Anguilla anguilla*) exported to the U.S.** Appendix-II export data from 2018-2021. Data accessed 01 June 2022 (<https://trade.cites.org>).

| Year | Importer | Exporter | Origin | Quantity (kg) |
| --- | --- | --- | --- | --- |
| 2018 | U.S. | China | Morocco | 21,000 |
| 2018 | U.S. | China | Unknown | 18,600 |
| 2019 | U.S. | - | - | 0 |
| 2020 | U.S. | China | Morocco | 265,500 |
| 2021 | U.S. | - | - | 0 |
